# Association of a Novel Homozygous Variant in *ABCA1* Gene with Tangier Disease

**DOI:** 10.3390/jcm12072596

**Published:** 2023-03-30

**Authors:** Sofía Barbosa-Gouveia, Silvia Fernández-Crespo, Héctor Lazaré-Iglesias, Arturo González-Quintela, Néstor Vázquez-Agra, Álvaro Hermida-Ameijeiras

**Affiliations:** 1 Fundación Instituto Investigación Sanitaria de Santiago de Compostela (FIDIS), University of Santiago de Compostela (USC), 15782 Coruña, Spain; 2 National Reference Centre for Inherited Metabolic Diseases, CIBERER, MetabERN, 28029 Madrid, Spain; 3 Department of Internal Medicine, University Hospital of El Bierzo, 24404 Ponferrada, Spain; 4 Department of Pathology, University Clinical Hospital of Santiago de Compostela, 15706 Santiago de Compostela, Spain; 5 Rare Diseases Center, Minority Diseases Working Group from the Spanish Society of Internal Medicine (GTEM), University Clinical Hospital of Santiago de Compostela, 15706 Santiago de Compostela, Spain

**Keywords:** ABCA1, Tangier disease, high-density lipoprotein, ApoA-I, bioinformatics

## Abstract

Tangier disease (TD) is a rare autosomal recessive disorder caused by a variant in the *ABCA1* gene, characterized by significantly reduced levels of plasma high-density lipoprotein cholesterol (HDL-C) and apolipoprotein A-1 (ApoA-I). TD typically leads to accumulation of cholesterol in the peripheral tissues and early coronary disease but with highly variable clinical expression. Herein, we describe a case study of a 59-year-old male patient with features typical of TD, in whom a likely pathogenic variant in the *ABCA1* gene was identified by whole-exome sequencing (WES), identified for the first time as homozygous (NM_005502.4: c.4799A>G (p. His1600Arg)). In silico analysis including MutationTaster and DANN score were used to predict the pathogenicity of the variant and a protein model generated by SWISS-MODEL was built to determine how the homozygous variant detected in our patient may change the protein structure and impact on its function. This case study describes a homozygous variant of the *ABCA1* gene, which is responsible for a severe form of TD and underlines the importance of using bioinformatics and genomics for linking genotype to phenotype and better understanding and accounting for the functional impact of genetic variations.

## 1. Introduction

Tangier disease (TD) is a rare autosomal recessive disorder caused by homozygous or compound heterozygous variants in the *ABCA1* gene which encodes a cell plasma membrane cholesterol transporter. The ATP-binding cassette (ABC) transporter superfamily is a class of transmembrane proteins that bind ATP and use its energy for the influx/efflux transport of several substrates across cell membranes, including lipids, cytotoxins, and metabolites [1,2,3]. The member of the subfamily *ABCA1* (ATP-binding cassette, subfamily A, member 1) encodes a protein that functions as a key gatekeeper of intracellular cholesterol transport allowing the regulation of cellular cholesterol and phospholipid efflux, which will then bind to apolipoprotein A-I (ApoA-I) for high-density lipoprotein (HDL) biosynthesis, transport of apolipoprotein E (ApoE), interleukins −1β, and molecule drugs [4,5,6,7]. *ABCA1* is highly expressed in several tissues such as placenta, pancreas, liver, intestine, heart, and lung [8,9,10,11,12,13]. It also participates in physiological and pathological processes such as dysregulation of lipid metabolism, cardiovascular diseases, inflammation, and cancer development [14,15,16,17]. Several models have proposed the special role of ABCA1 in cholesterol efflux; however, the detailed mechanism by which ABCA1 transports cholesterol to ApoA-1 is not yet fully understood [18]. So far, it is known that intracellular free cholesterol and phospholipids cross the plasma membrane to combine with apoproteins (mainly ApoA-I), forming HDL. This is the first step of reverse cholesterol transport, a process by which cholesterol excess is removed from peripheral tissues and distributed through the liver and other tissues and removed through the gallbladder (REF) [19]. TD is mainly characterized by accumulation of cholesterol in cells, particularly in the reticuloendothelial system, by high-density lipoprotein cholesterol (HDL-C) levels < 25 mg/dL and apolipoprotein A-1 (ApoA-I) < 20 mg/dL [20]. Classic *ABCA1* pathogenic variants are associated with most severe phenotypic presentations while heterozygous carriers may result in mitigated phenotypes with reduced HDL-C levels that are intermediate between Tangier disease and normal, so it is believed that it may be underdiagnosed [21]. Reduced reverse cholesterol transport leads to accumulation of cholesterol in the peripheral tissues, and to the major physical examination findings: hyperplastic yellow-orange tonsils, corneal opacities, enlarged liver, spleen, and lymph nodes, and peripheral neuropathy (sensory, motor, or mixed, temporary or permanent, mild or severe). 

Typical laboratory findings in Tangier disease include low blood platelet count, and almost undetectable plasma levels of low-density lipoprotein cholesterol (LDL-C) but normal or high plasma triglycerides [22]. The differential diagnosis includes LCAT (lecithin–cholesterol-acyltransferase) or ApoA-I deficiency (hypoalphalipoproteinemia) and hypo-HDL-cholesterolemia secondary to pharmacological treatment (probucol, fibrates). Currently, there is no curative treatment available. In this article, we sought to describe the pathogenicity of a homozygous variant in *ABCA1* in a patient with suspected Tangier disease for an accurate characterization of the disease-causing variant. In TD, is important to control cardiovascular risk factors in order to prevent the development of atherosclerosis and early coronary disease [23,24]. Regular screening for atherosclerotic coronary artery disease by stress echocardiography is recommended [25].

## 2. Experimental Section

### 2.1. Clinical Profile

A recently retired 59-year-old Spanish male with sedentary lifestyle and current smoking habit was referred to our institution because of unexplained hepatosplenomegaly. He did not drink alcohol. At that time, there was history of monoclonal gammopathy of undetermined significance (MGUS) involving IgG kappa and IgA lambda monoclonal cell populations not showing disease progression. Medical history also included chest trauma at 21 years old suffering multiple injuries, including pneumothorax and T11 burst fracture. The only medication he was taking was Escitalopram 10 mg. Patient provided written informed consent for the publication of the results. 

As regards family background, the patient’s parents both died at an elderly age, and three brothers had died as a result of premature cardiovascular death. They had also been diagnosed with demyelinating neuropathy, probably related to leprosy (but neither granuloma nor stains for acid-fast bacilli (AFB) were observed, just “bacillary degeneration”). They also showed altered lipid profile (low concentrations of ApoA-I and ApoB), thrombocytopenia, and splenomegaly (one of them underwent emergency splenectomy due to spontaneous splenic rupture and the histological examination revealed numerous foamy macrophages in the immunohistochemical analysis of splenic lymph nodes). One other sibling died of laryngeal cancer and the fifth one is still alive, diagnosed with the same variants in homozygous state and with a similar clinical presentation of low high-density lipoprotein cholesterol (HDL-C). The patient was admitted due to acute deterioration of liver function and splenectomy was performed to achieve prolonged normalization of his platelet count. He is currently fully recovered, and he is actually being monitored for presence of subclinical atherosclerotic lesions and under lipid-lowering agents (statins and omega-3 fatty acid formulations).

**Figure 1:**
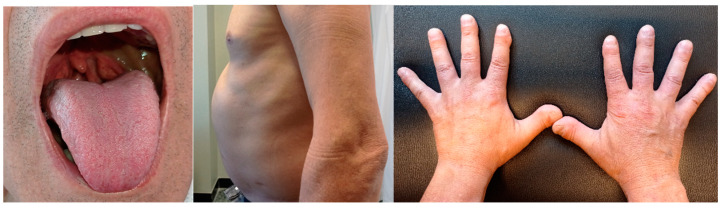
(**Left**): Orange tonsils. (**Middle**): Massive hepatosplenomegaly. (**Right**): Clubbing.

Physical examination: mild blood pressure elevation, BMI within the normal range, yellowing of sclera, orange-colored tonsils (Figure 1—left), red-colored and intensely itchy nodules on the nape of the neck (diagnosed as prurigo nodularis of Hyde), aortic and mitral stenosis murmurs, distended abdomen with massive hepatosplenomegaly (Figure 1—middle), umbilical hernia, vascular murmurs at both femoral arteries, clubbing (Figure 1—right) and absence of both Achilles tendon reflexes. The patient did not show any physical sign of dyslipidemia such as tendon xanthomas, xanthelasma palpebrarum, or corneal arcus. 

The laboratory data were as follows: total cholesterol (TC): 48 mg/dL (NR: 120–255), low-density lipoprotein cholesterol (LDL-C): 3 mg/dL (NR: 55–125), high-density lipoprotein cholesterol (HDL-C): 2 mg/dL (NR: 34–91), very-low-density lipoprotein cholesterol (VLDL-C): 42 mg/dL, triglycerides (TGC): 211 mg/dL (NR: 27–150), lipoprotein A 1 mg/dL, apolipoprotein A (ApoA-I): 0 mg/dL and apolipoprotein B (ApoB): 51 mg/dL. Further laboratory investigations of blood evidenced mild low red cell and platelet counts and elevation of transaminases, prolonged partial thromboplastin time (PTT), with high bilirubin and low albumin reflected liver dysfunction. The flow cytometric analysis and electrophoretic proteinogram were consistent with his condition of monoclonal gammopathy of unknown significance. Bone marrow biopsy extracted from the femur reflected foamy histiocytic infiltrates without fibrosis. Autoimmune and viral serologies and tumor markers were negative.

In the electrophysiological study, an amplitude asymmetry of the sensory response in both sural nerves was observed as well as bilateral L5/S1 fasciculations without any evidence of denervation.

Chest X-ray, electrocardiogram, ambulatory blood pressure monitoring, stress echocardiogram, and urine albumin/creatinine ratio tests were also performed, and results were unremarkable. An echocardiogram revealed mild tricuspid and mitral valve regurgitation with preserved left ventricular ejection fraction.

**Figure 2:**
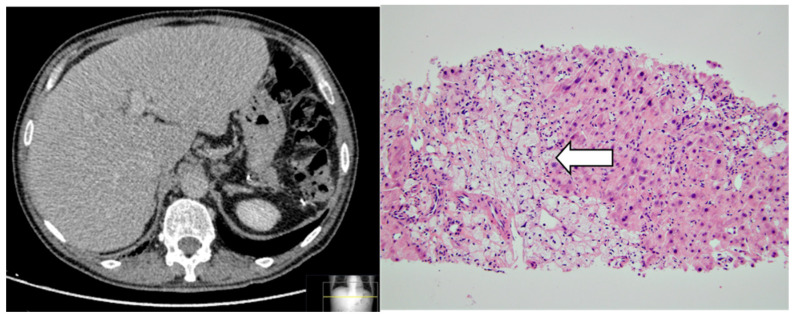
(**Left**): Abdominal computed tomography showing hepatomegaly. (**Right**): Hematoxylin and eosin (HE) staining of liver tissue (original magnification ×20): note the presence of clusters of foamy histiocytes involving liver tissue due to lipid deposition (white arrow).

Abdominal ultrasound and ulterior computed tomography (Figure 2—left) showed liver enlargement 17 cm (mainly caudate lobe and left lobe lateral segments). Contours were irregular suggesting chronic liver injury without any space-occupying lesion. Liver biopsy showed aggregates of histiocytes with foamy PAS-negative cytoplasm, diffusely distributed throughout the lobule, portal spaces, and centrilobular region (Figure 2—right). Some regenerative nodules surrounded by fibrosis were also reported. Upper GI endoscopy and antral biopsies evidenced chronic inflammation, but foamy histiocytes were absent (images not shown).

Secondary causes of hyperlipidemia were ruled out and we focused on assessing for rare hereditary metabolic disorders of lipid metabolism. 

### 2.2. Targeted Next-Generation Sequencing

Patient’s DNA was isolated from 400 μL of blood collected in EDTA following standard procedures and whole-exome sequencing (WES) was performed. Genomic DNA was enriched for exome sequencing using the Human Core Exome kit by Twist Bioscience (Sophia Genetics SA, Lausanne, Switzerland) following the manufacturer’s recommendations. Enriched libraries were sequenced on the NextSeq platform (Illumina Inc., San Diego, CA, USA) using a multiplex system with 16 samples per run with the NextSeq 500/550 Mid Output V2 kit (Illumina Inc., San Diego, CA, USA). Both variant annotation and filtering were performed using SOPHiA DDM^®^ software, version 4.7.5 (Sophia Genetics SA). The genetic variant calls were performed against the reference sequence of GRCh37/hg19 from the University of California Santa Cruz (UCSC) Genome Browser.

To ensure a reliable clinical interpretation of the variants detected, and to predict their pathogenicity, we considered prioritization criteria according to American College of Medical Genetics and Genomics (AMCG) guidelines [26]. We considered allele frequency using the Exome Aggregation Consortium database (ExAC) [27], 1000 Genomes Project database [28], and GnomAD [28]. Several pathogenicity algorithms were used such as MutationTaster, FATHMM (Functional Analysis through Hidden Markov Models), and DANN (Deleterious Annotation of genetic variants using Neural Networks) scores. Genomic Evolutionary Rate Profiling (GERP), PhyloP, and phastCons, were applied to assess variants located in highly conserved regions. 

### 2.3. Protein Modeling

The *ABCA1* gene encodes 254 kDa protein composed of 2261 amino acids. The ABCA1 (UniProt AC: A5D8U6; ID: A5D8U6_HUMAN) protein FASTA sequence was used to build a protein model with SWISS-MODEL. The SWISS-MODEL template library (SMTL version 28 February 2022, PDB released 28 February 2022 [29]) was searched with BLAST (Basic Local Alignment Search Tool) [30] and HHBlits (hidden Markov model (HMMs)-based lightning-fast iterative sequence search) [31] for evolutionary-related structures matching the target sequence. This homology model was used in order to investigate how the homozygous variant detected in our patient could alter the protein structure, potentially resulting in conformational changes with a significant impact on function (most likely loss of function). 

## 3. Results

### 3.1. Molecular Genetics and In Silico Analysis of the ABCA1 Variant

The NGS analysis allowed the identification of a homozygous missense variant, c.4799A>G (p. His1600Arg) in *ABCA1* gene (NM_005502.4). The identified *ABCA1* variant was analyzed in silico to predict pathogenicity and the functional consequences, as well as determine the degree of evolutionary conservation and minor allele frequency (Table S1). Pathogenicity was predicted using MutationTaster (disease-causing) and by calculating the DANN score (score: 0.9984) which corresponded to “damaging”. According to GERP, PhyloP, and phastCons, the variant c.4799A>G is placed in a highly conserved region of the ABCA1 protein. It was not cited on ClinVar, nor was it referenced in population databases such as GnomAD (exome and genome) or correlated with patient’s phenotype, and so it was, therefore, classified as “likely pathogenic” according to the following ACMG criteria: PM2, PM5, PP3, and PP4 [25]. The definitive diagnosis of Tangier disease was made considering the Koseki criteria [24].

### 3.2. AGK Protein Modeling

**Figure 3:**
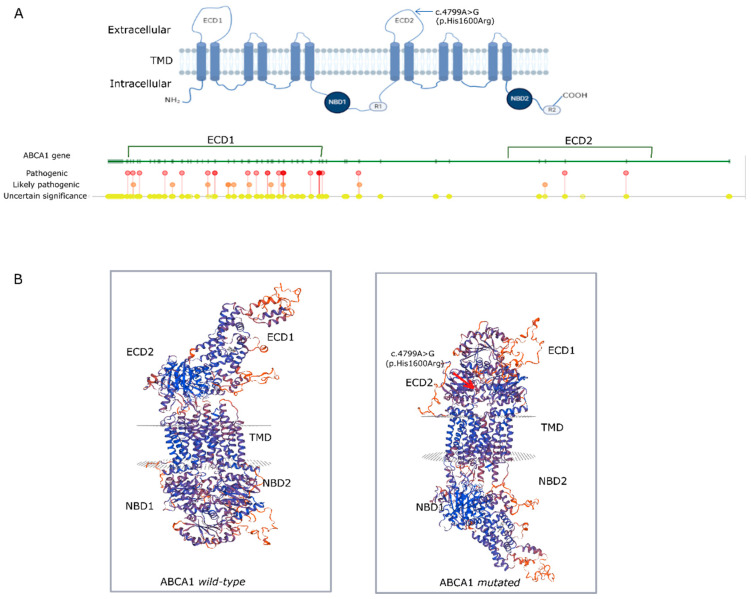
(**A**): Graphical model of the protein domains of ABCA1 along with the *ABCA1* gene representation with the variants identified in ClinVar. (**B**): Predicted dimensional model of the wild-type ABCA1 protein without the variant and the ABCA1 variant c.4799A>G (p. His1600Arg), highlighted with a red arrow, reflecting the functional consequences of single-point mutation by substitution of the polar amino acid histidine to the positively charged arginine. ECD—extracellular domain; TMD—transmembrane domains; NBD—nucleotide-binding domains; R1 and R2: regulatory domains.

The ABCA1 protein contains 2261 amino acids and comprises two transmembrane domains (TMD), two nucleotide-binding domains in the cytoplasm (NBD1 and NBD2), and two extracellular domains that are implicated in regulatory roles (R1 and R2). In addition, distinct from all the other ATP-binding cassette (ABC) transporters, the ABCA1 protein has two large extracellular domains, ECD1 and ECD2 (Figure 3A). The protein modelling of wild-type and mutated ABCA1 showed predicted changes in spatial protein structure (Figure 3B) affecting the structural conformation of ECD1 and ECD2 in the extracellular region.

## 4. Discussion

We report the case of a patient referred to our clinic presenting features typical of Tangier disease with a likely pathogenic *ABCA1* variant, c.4799A>G, identified for the first time as homozygous. Several types of variants in *ABCA1* have been identified, with missense and frameshift variants being the most often described [32]. In ClinVar, there are 37 likely pathogenic/pathogenic variants in *ABCA1,* of which 14 are associated with TD. It is worth noting the high number of functional single nucleotide polymorphisms (SNPs) identified through genome-wide association studies (GWAS) associated either with disease risk or protective role. Several SNPs in *ABCA1* have shown a strong association between plasma lipid levels and coronary heart disease susceptibility, while others were associated with a protective role [33,34]. Since the efflux of free cholesterol and phospholipids to ApoA-I is defective, TD patients usually have free-cholesterol (foam cells) overload in macrophages and other cells, which in turn are involved in the pathogenesis of atherosclerosis and coronary heart diseases (CHD). However, while CHD can develop at any age, reduced low-density lipoprotein cholesterol (LDL-C) in TD patients seems to provide cardiovascular protection, but those patients with normal LDL-C levels are likely to develop premature CHD [35].

According to a recent review from Mercan et al. [36], the patient presented the most usual clinical features of TD including splenomegaly (40.7%), the characteristic orange tonsils (33%), and thrombocytopenia (27.8%), in addition to very low concentrations of HDL-C. Less frequent findings such as hepatomegaly (13%), valve disease (13%), and anemia (9.3%) were also present. Regarding prurigo nodularis of Hyde, initially considered idiopathic, it has been also reported to be a manifestation of this disease in 14.8% of patients [9]. Regarding ocular manifestations of TD, corneal stromal opacities are commonly observed, peripheral and finely stippled, usually asymptomatic [37]. The slit-lamp examination of the patient here reported revealed crystalline powdery opacities affecting both eyes, which can be considered an atypical manifestation.

Histological lipid deposits in clusters of foamy histiocytes, such as those found in the liver of this patient, have been commonly reported in multiple tissues including tonsils, bone marrow, skin, and jejunal submucosa; Schwann cells in peripheral nerves and in nonvascular smooth muscle cells cannot be considered pathognomonic since they can be seen in a variety of different storage diseases [38,39,40]. The history of a sibling with reported spontaneous splenic rupture and numerous foamy macrophages in the immunohistochemical analysis of splenic lymph nodes is remarkable.

Considering the patient’s positive family history of coronary heart disease, thrombocytopenia, splenomegaly, and peripheral polyneuropathy, it seems reasonable to conclude those three siblings with unexpected sudden death could be also affected by TD, although this cannot be confirmed due to the lack of genetic testing. Premature coronary artery disease is frequently observed (up to 25% of patients with TD) [41]. Increased cardiovascular risk may be due to accelerated atherogenicity, increased intima–media thickness, or poor response to lipid-lowering therapies [42,43].

Neuropathic manifestations are very heterogeneous among individuals with TD, with distal symmetric polyneuropathy predominance but with no further reproducible patterns of functional alterations, age of onset, or a clear genotype–phenotype correlation [36]. Di Pasquale et al. suggest possible subtle abnormalities that escape quantitative high-resolution ultrasonography detection [44]. In this case study, three siblings were diagnosed with demyelinating neuropathy, probably related to leprosy, but in the case of this study, it might probably be a misdiagnosis. Both sural sensory nerves’ action potential amplitude asymmetry in this reported patient cannot be definitely linked to neuropathic manifestation of Tangier disease since he also suffers from lumbar degenerative disc disease. ABCA1 is a key transporter that mediates cholesterol efflux from cells and comprises two transmembrane domains (TMD) [45], two nucleotide-binding domains in the cytoplasm (NBD1 and NBD2), and two extracellular domains (R1 and R2). In addition, the ABCA1 protein has two large extracellular domains, ECD1 and ECD2 [46,47]. Disulfide bonds have been described within the ECD1 and ECD2 domains and both are essential for accommodating ApoA-I and generation of high-density lipoproteins [48]. Fasano et al. [38] reported a patient who was compound heterozygous for a nucleotide substitution of c.4799A>G (p. His1600Arg) which was considered probably damaging based on predictive in silico analysis. The patient here reported is found to be homozygous for the same variant, resulting in the conversion of the polar histidine amino acid to the positively charged arginine. The three-dimensional model generated with a SWISS-MODEL reinforces the pathogenic nature of the genetic variant identified since it may cause significant structural changes in the quaternary structure of ABCA1, leading to poor or inappropriate protein function and, therefore, increasing susceptibility to disease.

Activity of histidine residues can be modulated depending on protein interactions and it is a significant catalytic residue in many enzymes by shuttling protons and enhancing catalysis. ABCA1 mediates the delivery of phospholipids and cholesterol from the membrane to ApoA-I and p.His1600Arg is precisely located on the ECD2 domain which is responsible for binding to ApoA-II [49]. The patient here reported displayed undetectable plasma levels of ApoA-I.

In *ABCA1* molecular defects, the genotype–phenotype severity correlation is not often clear, since adult TD patients with homozygous or compound heterozygous variants do not always show all the clinical manifestations expected in TD. Some adult patients with nonsense variants can present CHD with yellow-orange tonsils or show peripheral sensory neuropathy with absent CHD [11]. According to this, it is reasonable to expect that besides the molecular defects in *ABCA1*, the clinical presentation of TD patients is also influenced by other factors such as transcriptional or posttranscriptional elements, other genes that participate in cholesterol efflux, nutritional factors, and age.

Indeed, *ABCA1* plays a critical role in maintaining cholesterol metabolism and HDL biosynthesis. Although the exact molecular mechanism is not yet fully understood, it is important to uncover the molecular defects to understand their mechanistic effects and develop new therapies. The findings from the present study expand the mutational spectrum of the *ABCA1* gene in Tangier disease and emphasize the important complement of whole-exome sequencing (WES) studies in establishing precise clinical diagnosis of this rare condition. Additionally, these results might shed light in the management of TD and develop targeted drugs that specifically regulate *ABCA1* expression.

## Data Availability

Data is contained within the article and supplementary material.

## Supplementary Materials

The following supporting information can be downloaded at: https://www.mdpi.com/article/10.3390/jcm12072596/s1, Table S1: In silico analysis to predict ABCA1 variant pathogenicity according to AMCG guidelines. Table S2: Clinical Features and Diagnostic Tests at Initial Presentation.
