# Supplementary material for: Association of a Novel Homozygous Variant in ABCA1 Gene with Tangier Disease
Source: J Clin Med. 2023 Mar 30;12(7):2596. doi: 10.3390/jcm12072596 (PMC10094818; doi:10.3390/jcm12072596)
Supplement: Supplementary file 1 [file jcm-12-02596-s001.zip › Supplementary Table S1.pdf]

Table S1. ***In silico*** **analysis to predict** ***ABCA1*** **variant pathogenicity according to AMCG guidelines**

**Variants *ABCA1* (NM_005502.4)**	**Inheritance**	**Population frequencies**			**Conservation Scores**			**Prediction Pathogenicity Scores**		
		**gnomAD**	**ExAC**	**1000 Genomes**	**GERP**	**PhyloP100way**	**PhastCons100way**	**DANN**	**Mutation Taster**	**FATHMM**
c.4799A>G	Mother Father	-	-	-	5.20	8.017	1.000	0.9984	DC	D

DC: Disease causing; D: Damaging; WT: Wild Type
