# Supplementary material for: Association of a Novel Homozygous Variant in ABCA1 Gene with Tangier Disease
Source: J Clin Med. 2023 Mar 30;12(7):2596. doi: 10.3390/jcm12072596 (PMC10094818; doi:10.3390/jcm12072596)
Supplement: Supplementary file 1 [file jcm-12-02596-s001.zip › Supplementary Table S2.pdf]

Table S2. *Clinical Features and Diagnostic Tests at Initial Presentation*

**Parameter**	**ABCA1 mutations [genotype], protein**	
	**[c.4799A>G], p. H1600R + [c.4799A>G], p. H1600R**	**[c.1758_1759insG], p.R587Afs*43 + [c.4799A>G], p.H1600R[a]**
**Clinical features**		
Age (years)	59	33
Sex	Male	Male
Motor neuropathy	Yes	Yes
Splenomegaly	Yes	Yes
Thrombocytopenia	Yes	Yes
Medical history of tonsillectomy	Yes	Yes
Orange-colored tonsils	Yes	No
Clubbing	Yes	No
Hypertelorism	No	Yes
Low set ears	No	Yes
Wide spaced nipples	No	Yes
Cutaneous manifestations	Prurigo nodularis of Hyde	Multiple freckles
Coronary heart disease	No	Yes
Monoclonal gammopathy of undetermined significance	Yes	No
**Biochemical analysis (units)**		
TC (mmol/L)	1.2	2.8
LDL-C (mmol/L)	0.07	1.2
TG (mmol/L)	2.3	3.2
HDL-C (mmol/L)	0.02	0.14
ApoB (g/L)	0.57	N/A
ApoAI (g/L)	Undetectable	<0.3
Foamy macrophages	Yes	Yes

[a]; patient reported by Fasano et al.[38] who was compound heterozygous for a nucleotide substitution c.4799A>G (p. His1600Arg)
